# Simulation of Left Ventricular Dynamics Using a Low-Order Mathematical Model

**DOI:** 10.1007/s13239-017-0327-9

**Published:** 2017-08-15

**Authors:** Michael J. Moulton, Brian D. Hong, Timothy W. Secomb

**Affiliations:** 1 Department of Surgery, Cardiothoracic Surgery, University of Nebraska Medical Center, 982315 Nebraska Medical Center, Omaha, NE 68198 USA; 2 Program in Applied Mathematics, University of Arizona, Tucson, AZ 85724 USA; 3 Department of Physiology, University of Arizona, Tucson, AZ 85724 USA

**Keywords:** Cardiac mechanics, Diastolic heart failure, Mathematical model, Myocardial strain, Pressure–volume curve, Ventricular wall stress

## Abstract

The eventual goal of this study is to develop methods for estimating dynamic stresses in the left ventricle (LV) that could be used on-line in clinical settings, based on routinely available measurements. Toward this goal, a low-order theoretical model is presented, in which LV shape is represented using a small number of parameters, allowing rapid computational simulations of LV dynamics. The LV is represented as a thick-walled prolate spheroid containing helical muscle fibers with nonlinear passive and time-dependent active contractile properties. The displacement field during the cardiac cycle is described by three time-dependent parameters, using a family of volume-preserving mappings based on prolate spheroidal coordinates. Stress equilibrium is imposed in weak form and the resulting force balance equations are coupled to a lumped-parameter model of the circulation, leading to a system of differential–algebraic equations, whose numerical solution yields predictions of LV pressure and volume, together with spatial distributions of stresses and strains throughout the cardiac cycle. When static loading of the passive LV is assumed, this approach yields displacement and stress fields that closely match results from a standard finite-element approach. When dynamic motion with active contraction is simulated, substantial variations of fiber stress and strain through the myocardium are predicted. This approach allows simulations of LV dynamics that run faster than real time, and could be used to determine patient-specific parameters of LV performance on-line from clinically available measurements, with the eventual goal of real-time, patient-specific analysis of cardiac parameters.

**Electronic supplementary material:**

The online version of this article (doi:10.1007/s13239-017-0327-9) contains supplementary material, which is available to authorized users.

## Introduction

The pumping function of the left ventricle (LV) depends not only on the active contractile force generated in its myocardial wall, but also on the size and shape of the LV, the thickness and passive mechanical properties of the wall, and the interaction of the LV with other parts of the circulatory system. Numerous theoretical models for LV mechanics have been developed for investigating normal LV function and the pathophysiology of diseased hearts, and for eventual application in patient-specific diagnosis and treatment planning.

One general class of models is based on geometrically detailed representations of cardiac anatomy, mechanics, electrophysiology and cardiac-circulatory system interactions.^11^,^13^,^17^,^20^,^28^ Typically, these models are analyzed using the finite element method, which uses a large number of degrees of freedom to represent cardiac deformation and other variables of interest. Such models have been used to estimate active and passive cardiac muscle parameters, by minimizing differences between predicted strains and experimental observations, for example from magnetic resonance imaging tagging.^3^,^32^ The computational cost of such models is generally high, and simulation of a single cardiac cycle may take hours or longer.^15^,^21^,^24^

Faster computational models for LV mechanics can be developed by restricting the number of degrees of freedom used to describe LV deformation. In this class of models, referred to here as “low-order,” the shape of the LV is represented approximately by a limited family of shapes, which can be specified by a small number of parameters. Generally, the shapes considered have rotational symmetry about the longitudinal axis of the LV. Examples of low-order models for LV dynamics include cylindrical models,^2^,^26^ spherical models^23^ and prolate spheroidal models.^4^,^25^,^31^ A low-order model that requires a rotationally symmetric LV shape^1^ is based on the assumption that muscle fiber stress and strain are approximately uniform throughout the myocardium. This model leads to the prediction that the ratio of fiber stress to LV internal pressure depends only on the ratio of LV wall volume to cavity volume, independent of LV shape. The varying elastance model,^36^ in which the mechanical characteristics of the pumping LV are represented by a single scalar equation, can be considered as an extreme of the low-order approach. This model is limited in that its parameters are not directly derived from mechanical characteristics of the myocardium and it does not include an explicit representation of myocardial stress and strain.

Advances in speckle tracking echocardiography have led to the availability of data on ventricular motion and deformation with excellent temporal resolution throughout the cardiac cycle. This opens up the possibility of deducing basic muscle parameters “on-line” in clinical settings^12^ based on ultrasound imaging. A system for patient-specific imaging and modeling, running on an echocardiography machine in a physician’s office or intensive care unit, would potentially allow physicians to assess cardiac muscle properties in real time, and to predict responses to therapeutic interventions. This would involve running multiple simulations of the cardiac cycle over ranges of parameters describing muscle properties to obtain the best fit to imaging data, and would therefore require that individual simulations run much faster than real time. For this purpose, low-order models as described above are more suitable than geometrically detailed models.

To be useful for this application, a low-order model should be able to approximately represent the shape and deformation of the LV through the cardiac cycle. In this regard, a prolate spheroidal model provides a better approximation than a cylinder or a sphere, because it includes the variation in wall curvature from base to apex. According to the law of Laplace, the generation of pressure is highly dependent on wall curvature. With regard to LV deformation, three main modes of deformation can be identified, namely base-to-apex shortening, circumferential contraction and torsion, and these should all be represented in the model. The myocardium is essentially incompressible and so its motions should be restricted to volume-preserving deformations. None of the models mentioned earlier meets all these criteria. For example, previous dynamic models based on prolate spheroidal geometry^4^,^25^ assumed a fixed relationship between the major and minor axes of the elliptical profile during deformation and therefore represent only a single mode of deformation.

As a step towards developing a model for LV dynamics that meets the requirements outlined above, we previously proposed a low-order model for LV kinematics,^27^ in which the LV is represented as a thick-walled, axisymmetric prolate spheroid that deforms according to a family of volume-preserving mappings defined by three time-dependent variables, and demonstrated the capability of the model to approximate the strain fields observed in the LV using speckle tracking echocardiography. A key aspect of that work was the development of volume-preserving mappings that represent three main modes of deformation, namely LV shortening, contraction and torsion, including nonlinear kinematic effects due to large strains.^19^ Here, we use this representation of LV kinematics by a small number of parameters as the basis for a low-order model of LV dynamics, and show that the model can be used to predict distributions of fiber stress and through the myocardium in computational simulations that run faster than real time.

## Methods

### Overview

The goal of the present study is to develop a low-order model for LV dynamics using this family of mappings, based on the theory of large-deformation continuum mechanics, including nonlinear active and passive material properties, viscoelasticity, and anisotropy with respect to muscle fiber orientation.^6^,^9^,^13^,^18^ A system of helical cardiac muscle fibers is introduced within the wall of the prolate spheroid, with fiber angle varying through the wall. The fiber angle and fiber strain are derived as functions of the kinematic parameters. The passive properties of the myocardium are represented by a Kelvin–Voigt type viscoelastic model. The elastic component is assumed to be transversely isotropic with respect to the fiber direction and is represented using a strain-energy function. The viscous component is characterized by a linear dependence on strain rate. Active fiber stress is dependent on a prescribed time-dependent activation function, and takes into account the length–tension and force–velocity characteristics of cardiac muscle, and the dependence of force generation on end-diastolic strain (Frank–Starling effect). The equations of stress equilibrium are solved approximately in weak form. The resulting equations are coupled to a lumped-parameter model of the circulatory system. This allows the dynamic behavior of the LV and its interaction with the circulatory system to be represented by a system of differential–algebraic equations, which can be solved rapidly by standard methods.

### Coordinate Systems

Prolate spheroidal coordinates (*μ*,*ν*,*ϕ*) are used with a time-dependent interfocal distance 2*a*. Then

1 $$ \begin{aligned} x = a\sinh \mu \sin \nu \cos \phi \hfill \\ y = a\sinh \mu \sin \nu \sin \phi \hfill \\ z = a\cosh \mu \cos \nu , \hfill \\ \end{aligned} $$

where (*x*,*y*,*z*) are Cartesian coordinates. The scale factors are $g_{\mu } = g_{\nu } = a\sqrt {\sinh^{2} \mu + \sin^{2} \nu }$ and $g_{\phi } = a\sinh \mu \sin \nu$ and the Jacobian is

2 $$ {\mathbf{J}} = \left[ {\begin{array}{*{20}l} {\partial x/\partial \mu } & {\partial x/\partial \nu } & {\partial x/\partial \phi } \\ {\partial y/\partial \mu } & {\partial y/\partial \nu } & {\partial y/\partial \phi } \\ {\partial z/\partial \mu } & {\partial z/\partial \nu } & {\partial z/\partial \phi } \\ \end{array} } \right] = \left[ {\begin{array}{*{20}c} {a\cosh \mu \sin \nu } & {a\sinh \mu \cos \nu } & 0 \\ 0 & 0 & {a\sinh \mu \sin \nu } \\ {a\sinh \mu \cos \nu } & { - a\cosh \mu \sin \nu } & 0 \\ \end{array} } \right], $$

where *ϕ* = 0 without loss of generality. The determinant is

3 $$ \left| {\mathbf{J}} \right| = a^{3} \sinh \mu \sin \nu \;(\sinh^{2} \mu + \sin^{2} \nu ). $$

**Figure 1:**
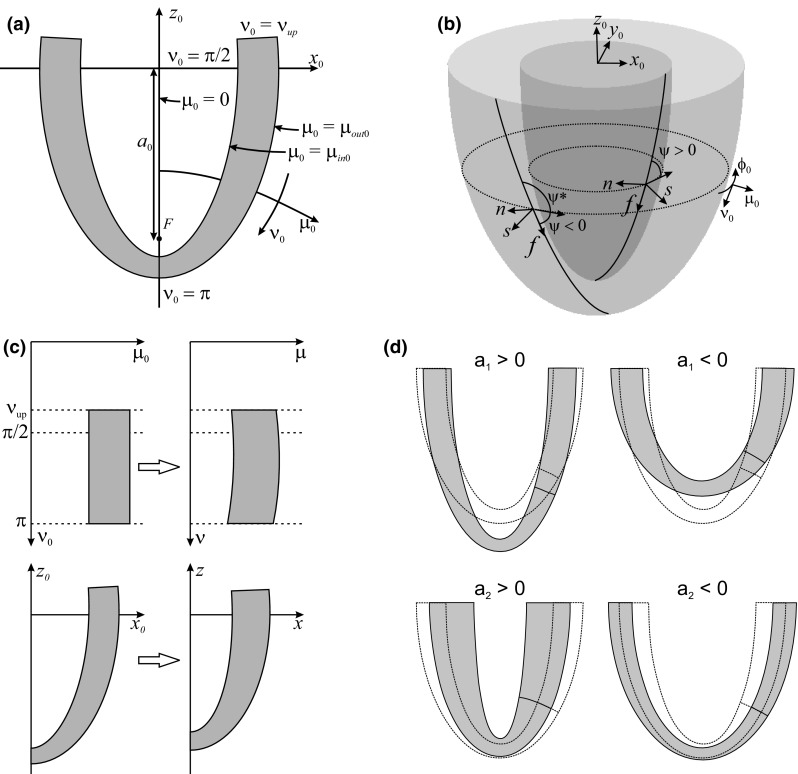
Coordinates and variables used to describe the geometry and deformation of the left ventricle (LV). (**a**) Section of reference LV shape in plane containing axis of rotational symmetry, showing Cartesian coordinates $(x_{0} ,y_{0} ,z_{0} )$ and prolate spheroidal coordinates $(\mu_{0} ,\nu_{0} ,\phi_{0} )$. The initial wall shape is characterized by interfocal distance $(2a_{0} )$, inner boundary $\mu_{0} = \mu_{in0}$, outer boundary $\mu_{0} = \mu_{out0}$ and upper edge $\nu_{0} = \nu_{up}$. (**b**) Three-dimensional representation of reference configuration, showing Cartesian coordinates $(x_{0} ,y_{0} ,z_{0} )$ and prolate spheroidal coordinates $(\mu_{0} ,\nu_{0} ,\phi_{0} )$. The local fiber coordinates $(s,n,f)$ and the fiber angles $\psi$ and $\psi^{ * }$ are shown for two helical fibers, one epicardial and one endocardial. For the epicardial fiber, $\psi$ < 0, and $\psi^{ * } = \psi + \pi$. For the endocardial fiber, $\psi$ > 0 and $\psi^{ * } = \psi$. (**c**) Schematic diagram of mapping from reference to deformed LV shape. Points in the $\mu_{0} - \nu_{0}$ plane are mapped to new $\mu$ values with no change in $\nu$. In the physical $x - z$ plane, the LV shape is deformed. (**d**) Effects of variations in parameters *a*_1_ and *a*_2_ on LV shape. In each case, the reference shape is shown by dashed curves and the deformed shape is indicated by the shaded region.

A reference configuration is defined with *a* = *a*_0_ and coordinates (*μ*_0_,* ν*_0_, *ϕ*_0_), in which the LV wall occupies the region $\mu_{in 0} \le \mu_{0} \le \mu_{out 0}$ and $\nu_{up} \le \nu_{0} \le \pi$ (Figs. 1a and 1b).

### Volume-Preserving Deformation

The deformation of the LV wall is represented using a family of mappings (*a*_0_;*μ*_0_,*ν*_0_,*ϕ*_0_) → (*a*;*μ*,*ν*,*ϕ*) that approximates the main modes of deformation. Volume preservation is ensured if volume elements in the initial and final configurations are equal:

4 $$ \left| {\mathbf{J}} \right|d\mu \,d\nu \,d\phi = \left| {{\mathbf{J}}_{0} } \right|d\mu_{0} \,d\nu_{0} \,d\phi_{0} , $$

where **J**_0_ is the Jacobian in the reference configuration. The assumption of axisymmetry implies that $d\phi = d\phi_{0}$. According to our previous method,^27^ we further assume that $\nu = \nu_{0}$ and obtain the mapping defined by:

5 $$ a^{3} \left[ {\cosh \mu \left(\frac{1}{3}\cosh^{2} \mu - \cos^{2} \nu_{0} \right) - \left(\frac{1}{3} - \cos^{2} \nu_{0}\right)} \right] = a_{0}^{3} \cosh \mu_{0} \left(\frac{1}{3}\cosh^{2} \mu_{0} - \cos^{2} \nu_{0} \right) - a_{2} a_{0}^{2} \cosh \mu_{in0} \left(\frac{1}{3}\cosh^{2} \mu_{in0} - \cos^{2} \nu_{0} \right) - a_{0}^{2} (a_{0} - a_{2} )\left(\frac{1}{3} - \cos^{2} \nu_{0} \right), $$

6 $$ \phi = \phi_{0} + a_{3} \,(\cos \nu_{0} - \cos \nu_{up} ), $$

where $a = a_{0} + a_{1}$. The mapping in the $\mu_{0} - \nu_{0}$ plane is illustrated in Fig. 1c. The first kinematic parameter gives lengthening (*a*_1_ > 0) or shortening (*a*_1_ < 0) of the LV at almost constant volume. The second parameter gives contraction (*a*_2_ > 0) or expansion (*a*_2_ < 0) (Fig. 1d). The third parameter gives torsional deformation that increases from base to apex and is clockwise (*a*_3_ < 0) or counterclockwise (*a*_3_ > 0) as viewed from the apex. The deformation gradient tensor **F**, the right Cauchy–Green strain tensor ${\mathbf{C}} = {\mathbf{F}}^{T} {\mathbf{F}}$ and the Green–Lagrange strain tensor ${\mathbf{E}} = {\frac{1}{2}}\left( {{\mathbf{C}} - {\mathbf{I}}} \right)$ can be computed in terms of the kinematic parameters^27^ (S10–15, equations labeled S refer to Supplementary Material). The dependence on $\cos \nu_{0}$ in (6) avoids a singularity in the deformation field at the apex. Equation (5) implicitly defines $\mu$ as a function of both $\mu_{0}$ and $\nu_{0}$. The dependence on $\nu_{0}$ implies that the deformed inner and outer LV surfaces are not generally spheroidal.

### Muscle Fiber Geometry

In the reference configuration, muscle fibers are assumed to lie in surfaces of constant $\mu_{0}$ $(\mu_{in0} \le \mu_{0} \le \mu_{out0} )$, and are identified by $\mu_{0}$ and by their angular position $\phi_{b}$ $(0 \le \phi_{b} \le 2\pi )$ at the base of the heart. Position on a given fiber is parameterized by $\nu_{0}$ $(\nu_{up} \le \nu_{0} \le \pi )$. The helical arrangement of the fibers and the variation of the helix angle through the wall are represented by setting $\phi_{0} = \phi_{b} + \omega (\cos \nu_{0} - \cos \nu_{up} )$, where $\omega$ is a $\mu_{0}$-dependent wrapping parameter. As indicated in Fig. 1b, the orientation of the fibers relative to the $\phi$-coordinate direction is measured either by $\psi$ $( - \pi /2 < \psi \le \pi /2)$ or by $\psi^{ * }$ $(0 \le \psi^{ * } < \pi )$, where

7 $$ \psi^{ * } = \left\{ {\begin{array}{ll} {\pi + \psi }; & {\psi < 0} \\ {\psi }; & {\psi \ge 0} \\ \end{array} } \right.. $$

The angle $\psi^{ * }$ is related to $\omega$ by (S23):

8 $$ \cos \psi^{ * } = \frac{{\omega \sinh \mu_{0} \sin^{2} \nu_{0} }}{{\sqrt {\sinh^{2} \mu_{0} + \sin^{2} \nu_{0} + \omega^{2} \sinh^{2} \mu_{0} \sin^{4} \nu_{0} } }}. $$

Streeter *et al*.^35^ found that the fiber angle $\psi = \psi_{eq}$ at the equator of the heart $(\nu_{0} = \pi /2)$ varies from $\psi_{in} = 85^{ \circ }$ on the inner wall to $0^{ \circ }$ at the midwall and to $\psi_{out} = - 65^{ \circ }$ on the outer wall. This is represented assuming linear variation with $\mu_{0}$, according to

9 $$ \psi_{eq} = \psi_{in} \frac{{\mu_{out} - \mu_{0} }}{{\mu_{out} - \mu_{in} }} + \psi_{out} \frac{{\mu_{0} - \mu_{in} }}{{\mu_{out} - \mu_{in} }}. $$

When $$ \nu_{0} = \pi /2 $$, (8) gives

10 $$ \cos \psi_{eq}^{ * } = \frac{{\omega \sinh \mu_{0} }}{{\sqrt {\cosh^{2} \mu_{0} + \omega^{2} \sinh^{2} \mu_{0} } }} $$

which can be solved for the wrapping parameter,

11 $$ \omega = \cot \psi_{eq}^{ * } \coth \mu_{0} \;{\text{if}}\;\psi_{eq}^{ * } \ne 0. $$

A local coordinate system $(s,n,f)$ is defined based on the reference fiber geometry, where $f$ is the fiber direction, $s$ is the transmural direction and $n$ is perpendicular to $f$ and $s$ (Fig. 1b, S16–22). In the deformed geometry, the fiber paths are:

12 $$ \begin{aligned} x\left( {\mu_{0} ,\phi_{b} ;\nu_{0} } \right)& = a\sinh \mu \sin \nu_{0} \cos [\phi_{b} + (\omega + a_{3} )(\cos \nu_{0} - \cos \nu_{up} )] \hfill \\ y\left( {\mu_{0} ,\phi_{b} ;\nu_{0} } \right)& = a\sinh \mu \sin \nu_{0} \sin [\phi_{b} + (\omega + a_{3} )(\cos \nu_{0} - \cos \nu_{up} )] \hfill \\ z\left( {\mu_{0} ;\nu_{0} } \right)& = a\cosh \mu \cos \nu_{0} \hfill \\ \end{aligned} $$

and the increment of arc length along the fiber is given by:

13 $$ ds = a\sqrt {(1 + (\partial \mu /\partial \nu_{0} )^{2} )(\sinh^{2} \mu + \sin^{2} \nu_{0} ) + (\omega + a_{3} )^{2} \sinh^{2} \mu \sin^{4} \nu_{0} } d\nu_{0} , $$

where $\partial \mu /\partial \nu_{0}$ is given by (S7). In the reference configuration,

14 $$ ds_{0} = a_{0} \sqrt {\sinh^{2} \mu_{0} + \sin^{2} \nu_{0} + \sinh^{2} \mu_{0} \sin^{4} \nu_{0} \,\omega^{2} } d\nu_{0} . $$

The fiber stretch ratio is $\lambda_{f} = ds/ds_{0} = L_{s} /L_{s0}$ where *L*_*s*_ is the current sarcomere length and *L*_*s0*_ is the length of the sarcomere in the reference configuration. When $\psi_{eq} = 0$, $\omega$ is undefined and the fibers are parameterized instead by:

15 $$ \begin{aligned} x\left( {\mu_{0} ,\nu_{0} ;\phi } \right) = a\sinh \mu \sin \nu_{0} \cos \phi \hfill \\ y\left( {\mu_{0} ,\nu_{0} ;\phi } \right) = a\sinh \mu \sin \nu_{0} \sin \phi \hfill \\ z\left( {\mu_{0} ,\nu_{0} } \right) = a\cosh \mu \cos \nu_{0} . \hfill \\ \end{aligned} $$

### Active Fiber Stress

The Cauchy stress generated by active fiber contraction is uniaxial in the fiber direction and experimentally has a well-defined zero tension sarcomere length and a length at which maximum tension is generated.^37^ We approximate it by:

16 $$ \sigma_{ff} = A\left( t \right)G\left( {L_{s} } \right)\left( {F_{ 0} + k_{av} d\varepsilon_{f} /dt} \right)(1 + k\varepsilon_{f,ed} ), $$

17 $$ G\left( {L_{s} } \right) = \exp \left( {\frac{{ - \left( {L_{s} - L_{s\hbox{max} } } \right)^{2} }}{{2L_{sw}^{2} }}} \right). $$

Here, $F_{0}$ gives the magnitude of the stress, $\varepsilon_{f} = {\frac{1}{2}}\left( {\lambda_{f}^{2} - 1} \right)$ is the fiber strain, $k_{av}$ defines the force–velocity characteristics of the fibers and $k$ determines the sensitivity of active force to preload according to the Frank–Starling mechanism, where $\varepsilon_{f,ed}$ is the end-diastolic fiber strain, $L_{s\hbox{max} }$ is the length of the sarcomere at which maximum tension is generated and $L_{sw}$ determines the width of the peak in the length–tension curve. The time-dependent fiber activation is given by

18 $$ A\left( t \right) = \left\{ {\begin{array}{*{20}l} {[\sin (\pi {\kern 1pt} t/T_{a} )]^{d} \;{\text{where}}\;d = 1/(1 + k^{\prime}\varepsilon_{f,ed} )\;{\text{when}}\; 0\le t \le T_{a} } \hfill \\ {0\;\quad {\text{when}}\;T_{a} \le t \le T_{c} ,} \hfill \\ \end{array} } \right. $$

where $t$ is time after the start of contraction in a given cycle, $T_{a}$ is the period of activation and $T_{c}$ is the period of the cardiac cycle. The exponent $d$ steepens the rise and fall of activation with increasing end-diastolic strain $\varepsilon_{f,ed}$, according to the observation that activation is prolonged with increased preload.^10^,^34^ The parameter $k^{\prime}$ defines the sensitivity of the activation function to preload. Since $\varepsilon_{f,ed}$ varies with position in the myocardium, $A\left( t \right)$ is a function of position. The resulting Cauchy stress is ${\varvec{\upsigma}}_{f} = \sigma_{ff} {\mathbf{e}}_{f} \otimes {\mathbf{e}}_{f}$ where $\otimes$ denotes the tensor product and ${\mathbf{e}}_{f}$ is the basis vector for the local fiber direction. The corresponding second Piola–Kirchhoff (PK2) stress^33^ is ${\mathbf{S}}_{f} = \lambda_{f}^{ - 2} \sigma_{ff} {\mathbf{e}}_{f} \otimes {\mathbf{e}}_{f}$.

### Passive Material Properties of Myocardium

The passive myocardium is represented as an incompressible anisotropic viscoelastic solid,^6^,^39^ using a Kelvin solid model for viscoelasticity. The Cauchy stress is $- p_{M} {\mathbf{I}} + {\varvec{\upsigma}}_{f} + {\varvec{\upsigma}}_{e} + {\varvec{\upsigma}}_{v}$, where $- p_{M} {\mathbf{I}}$ is the reaction stress due to the constraint of incompressibility^30^ and the corresponding PK2 stress is $- p_{M} {\mathbf{C}}^{ - 1}$.^33^ The elastic stress ${\varvec{\upsigma}}_{e}$ is assumed to be transversely isotropic with respect to the fibers^14^ and given by a strain-energy function^9^,^13^

19 $$ \varPsi = {\frac{1}{2}}c_{1} \left( {e^{W} - 1} \right) $$

where

20 $$ W = b_{ff} E_{ff}^{2} + b_{xx} \left( {E_{nn}^{2} + E_{ss}^{2} + E_{sn}^{2} + E_{ns}^{2} } \right) + b_{fx} \left( {E_{fn}^{2} + E_{nf}^{2} + E_{fs}^{2} + E_{sf}^{2} } \right). $$

$E_{ij}$ are the Green–Lagrange strain components in the fiber coordinates $(s,n,f)$ and $c_{1} ,b_{ff} ,b_{xx} ,b_{fx}$ are material parameters. The PK2 stress corresponding to ${\varvec{\upsigma}}_{e}$ is

21 $$ {\mathbf{S}}_{e} = c_{1} e^{W} \left[ {\begin{array}{*{20}c} {b_{xx} E_{ss} } & {b_{xx} E_{sn} } & {b_{fx} E_{sf} } \\ {b_{xx} E_{ns} } & {b_{xx} E_{nn} } & {b_{fx} E_{nf} } \\ {b_{fx} E_{fs} } & {b_{fx} E_{fn} } & {b_{ff} E_{ff} } \\ \end{array} } \right]. $$

The viscous stress ${\varvec{\upsigma}}_{v}$ is defined analogously to that in a viscous fluid^30^

22 $$ {\varvec{\upsigma}}_{v} = k_{vm} \left( {\nabla {\mathbf{v}} + \nabla {\mathbf{v}}^{T} } \right), $$

where **v** is the material velocity field and $k_{vm}$ is a constant. The corresponding PK2 stress is (S32):

23 $$ {\mathbf{S}}_{v} = k_{vm} {\mathbf{C}}^{ - 1} {\dot{\mathbf{C}}\mathbf{C}}^{ - 1} . $$

The components of ${\mathbf{S}}_{f}$ and ${\mathbf{S}}_{e}$ are converted from fiber coordinates to prolate spheroidal coordinates using the rotation matrix (S25):

24 $$ {\mathbf{Q}} = \left[ {\begin{array}{*{20}c} 1 & 0 & 0 \\ 0 & { - \cos \psi^{ * } } & {\sin \psi^{ * } } \\ 0 & { - \sin \psi^{ * } } & { - \cos \psi^{ * } } \\ \end{array} } \right]. $$

The total PK2 stress is ${\mathbf{S}} = - p_{M} {\mathbf{C}}^{ - 1} + {\mathbf{S}}_{f} + {\mathbf{S}}_{e} + {\mathbf{S}}_{v}$. Because the mappings are volume-conserving, the reaction stress does no work and can be omitted from the equilibrium equations.

### Equilibrium Equations

The equations of force equilibrium are expressed in weak form.^5^ The total virtual work done by an incremental displacement is zero,

25 $$ \iiint\limits_{{\Omega_{0} }} {d{\mathbf{E}}^{T} :{\mathbf{S}}dV_{0} } = \iint\limits_{{\partial \Omega_{e} }} {d{\mathbf{u}}_{e} \cdot {\mathbf{T}}_{e} dS} + \iint\limits_{{\partial \Omega_{b} }} {d{\mathbf{u}}_{b} \cdot {\mathbf{T}}_{b} dS}, $$

where ${\mathbf{T}}_{e}$ and ${\mathbf{T}}_{b}$ are the boundary tractions generated by the LV pressure on the LV endocardium and the myocardial boundary at the base of the heart, $dV_{0}$ is a differential volume element of the reference configuration $\Omega_{0}$, and $\partial \Omega_{e}$ and $\partial \Omega_{b}$ are the endocardial surface and the myocardial boundary at *ν* = *ν*_*up*_ in the current configuration $\Omega$. The incremental displacements and strains are defined by changes in the kinematic parameters, *i.e.*, $d{\mathbf{u}} = (\partial {\mathbf{u}}/\partial a_{i} )da_{i}$ where **u** is the displacement and $d{\mathbf{E}} = (\partial {\mathbf{E}}/\partial a_{i} )da_{i}$ (S33–55), and (25) gives:

26 $$ \iiint\limits_{{\Omega_{0} }} {\frac{{\partial {\mathbf{E}}^{T} }}{{\partial a_{i} }}:{\mathbf{S}}dV_{0} } = \iint\limits_{{\partial \Omega_{e} }} {\frac{{\partial {\mathbf{u}}_{e} }}{{\partial a_{i} }} \cdot {\mathbf{T}}_{e} dS_{e} } + \iint\limits_{{\partial \Omega_{b} }} {\frac{{\partial {\mathbf{u}}_{b} }}{{\partial a_{i} }} \cdot {\mathbf{T}}_{b} dS_{b} }\;{\text{for}}\;i = 1,2,3. $$

Due to axisymmetry, the LHS (internal virtual work) reduces to an integral over a rectangular region in the $\mu_{0} - \nu_{0}$ plane (Fig. 1c), which is evaluated using 2D Simpson’s rule on a regularly spaced 9 × 11 rectangular mesh. The first term of the RHS (external virtual work) reduces to a line integral over $\nu_{0}$ (S65). The second term can be computed as the virtual work done on the upper boundary of the cavity (S67). These quantities are calculated at each time point using the current values of $a_{i}$. All terms in (26) depend nonlinearly on $a_{i}$, and ${\mathbf{S}}_{v}$ also depends linearly on $da_{i} /dt$ (S68–74). Therefore, (26) leads to a system of coupled ordinary differential equations (S75) for $a_{i} (t)$.

### Ventricular Volume

The LV cavity volume $V(t)$ is computed as a solid of revolution about the $z$ axis and its time derivative is (S76–78):

27 $$ \frac{\partial V}{\partial t} = \frac{\partial V}{{\partial a_{1} }}\frac{{da_{1} }}{dt} + \frac{\partial V}{{\partial a_{2} }}\frac{{da_{2} }}{dt}. $$

### Model of Circulatory System

**Figure 2:**
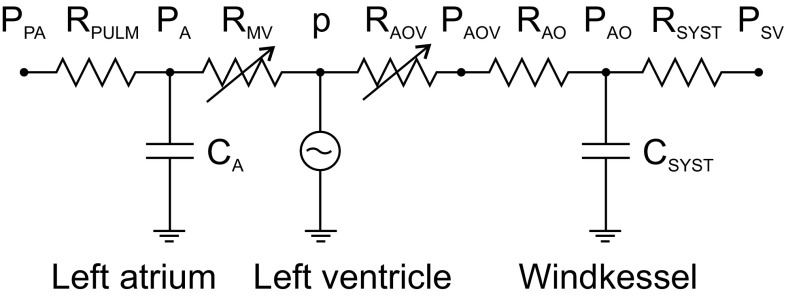
Schematic diagram of circulatory model indicating resistances, pressures and compliances.

The LV is coupled to a lumped-parameter model of the circulation (Fig. 2), including pulmonary vascular bed, left atrium, aorta and systemic vascular bed. Effects of LV inertia have been shown to be negligible and are not included.^7^,^29^ The mitral and aortic valves are represented by resistances $R_{MV}$ and $R_{AOV}$ that vary depending on the pressure differences across the valves:

28 $$ R_{MV} = R_{cc} - \frac{{R_{cc} - R_{op_mv} }}{{1 + e^{{ - \beta \left( {P_{A} - p} \right)}} }}, $$

29 $$ R_{AOV} = R_{cc} - \frac{{R_{cc} - R_{op_aov} }}{{1 + e^{{ - \beta \left( {p - P_{aov} } \right)}} }}, $$

where $R_{cc}$ is the resistance of a closed valve, $R_{op_mv}$ and $R_{op_aov}$ are the resistances of the open mitral and aortic valves, $P_{A}$ is the atrial pressure, $P_{aov}$ is the most proximal aortic pressure and $\beta$ is a constant. The proximal aortic pressure $P_{aov}$ is given by

30 $$ P_{aov} = \frac{{p \cdot R_{AO} + P_{AO} \cdot R_{AOV} }}{{R_{AO} + R_{AOV} }}, $$

where $R_{AO}$ is the proximal aortic resistance (impedance) and $P_{AO}$ is the aortic pressure after the impedance. Then the differential equations describing the interaction of the LV mechanics with the pulmonary, atrial and systemic circulation are given by:

31 $$ \frac{{dP_{A} }}{dt} = \frac{1}{{C_{A} }}\left( {\frac{{P_{PA} - P_{A} }}{{R_{PULM} }} - \frac{{P_{A} - p}}{{R_{MV} }}} \right), $$

32 $$ \frac{{dP_{AO} }}{dt} = \frac{1}{{C_{SYST} }}\left( {\frac{{P_{AOV} - P_{AO} }}{{R_{AO} }} - \frac{{P_{AO} - P_{SV} }}{{R_{SYST} }}} \right), $$

33 $$ \frac{\partial V}{{\partial a_{1} }}\frac{{da_{1} }}{dt} + \frac{\partial V}{{\partial a_{2} }}\frac{{da_{2} }}{dt} - \frac{{P_{A} - p}}{{R_{MV} }} + \frac{{p - P_{AOV} }}{{R_{AOV} }} = 0, $$

where $P_{PA}$ is the mean pressure in the pulmonary bed, $R_{PULM}$ is the resistance of the pulmonary circulation and $C_{A}$ is the compliance of the atrium.

### Numerical Solution Method

The force balance Eq. (26), together with (28–33), make up a system of differential–algebraic equations. The solution is obtained through a custom algorithm that solves the time integration using second order Runge–Kutta and solves for the solution vector at each time point using the Newton–Raphson method (S76–80). The resulting system is stiff during rapid valve resistance changes (valve openings and closings) and the time step was varied accordingly.

### Echocardiography Image Acquisition and Establishment of the Reference Configuration

A normal volunteer was studied with echocardiography. A standard echocardiography protocol was employed as described by Moulton and Secomb.^27^ Three long-axis (AP4, AP3 and AP2 views) echocardiography images obtained from an end-systolic echo frame were used to establish a reference configuration of the LV. A prolate spheroidal reference shape was obtained by minimizing the sum of squared deviations between the fitted points on the endocardial and epicardial surfaces and corresponding observed points in three long-axis images.^27^ The echo frame with lowest LV volume was chosen as the reference frame, given that an unloaded reference configuration is not identified by clinical imaging data.

### Construction of Models to Test Effects of LV Shape

Arts *et al*.^1^ derived an equation for the ratio of fiber stress *σ*_*ff*_ to cavity pressure *p* as a function of the ratio of wall volume *V*_*wall*_ to cavity volume ratio *V*:

34 $$ \frac{p}{{\sigma_{ff} }} = \frac{1}{3}\ln {\kern 1pt} \,\left( {1 + \frac{{V_{wall} }}{V}} \right). $$

**Figure 3:**
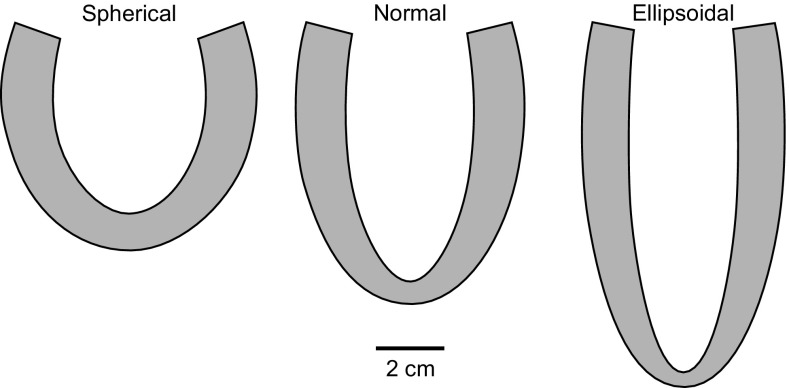
LV wall shapes used to examine effects of shape on stress distribution. All three shapes have the same cavity volume, 59.8714 cm^3^, and wall volume, 158.112 cm^3^. Parameter values are given in Table 1.

This derivation applies to any rotationally symmetric shape, under the assumption that the stress at each point in the wall is the sum of a hydrostatic pressure and a uniform unidirectional fiber stress. To test the predictions of this equation, we constructed three different LV models with identical wall volumes and cavity volumes, as follows: *normal*, with the shape derived from an image of a normal volunteer in early diastole; *spherical*, with a more spherical shape; *ellipsoidal*, with a more prolate shape. The three shapes are shown in Fig. 3 and model parameters are given in Table 1.

**Table 1:** Geometric parameters and work done for the three LV shapes. Work per cardiac cycle is averaged over twelve cycles.

	Spherical	Normal	Ellipsoidal
$a_{0}$ (cm)	2.7307	4.6307	6.7307
$\mu_{in}$	0.7470308	0.39521852	0.235
$\mu_{out}$	1.1173771	0.6719001	0.426
$\nu_{up}$	1.1112	1.1112	1.1112
$V$ (LV volume) (cm^3^)	59.8714	59.8714	59.8714
$V_{Wall}$ (LV wall volume) (cm^3^)	158.112	158.112	158.112
Fiber work/cycle (J)	1.039	1.004	0.982
Viscous work/cycle (J)	−0.105	−0.089	−0.082
Internal work/cycle (J)	0.933	0.915	0.900
External work/cycle (J)	0.933	0.915	0.900
Stroke work/cycle (J)	0.890	0.900	0.897

## Results

### Comparison with Finite Element Solution

**Figure 4:**
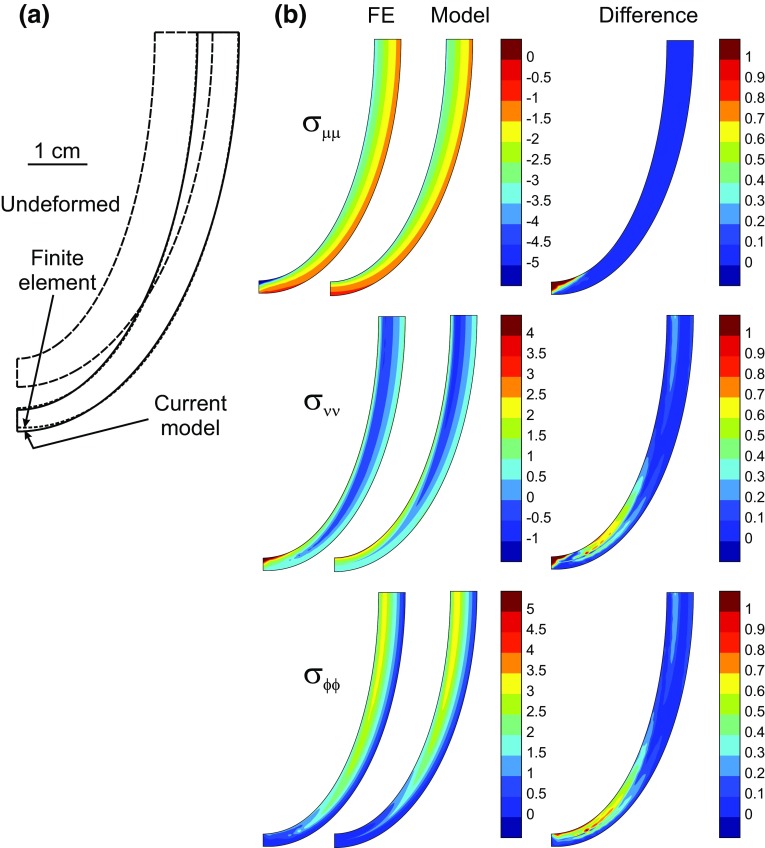
Comparison of current model and finite element solutions. (**a**) Undeformed configuration and deformed configurations computed by current model and by FE method. (**b**) Deviatoric normal stress components *σ*_*μμ*_, *σ*_*νν*_ and *σ*_*ϕϕ*_ computed by current model and by FE method, and magnitude of difference. Color bar scales in kPa.

Finite element (FE) simulations were performed using FlexPDE Version 3.11 (PDE Solutions Inc., Spokane Valley, WA 99206) for passive prolate spheroidal shells with nonlinear transversely isotropic material properties, statically loaded by internal pressure, and compared to corresponding simulations with the same loads, material properties and reference shape using the low-order method. A reference configuration was used with initial parameters *a*_0_ = 5.0, *μ*_in_ = 0.45, *μ*_out_ = 0.62, *ν*_up_ = π/2, yielding a cavity volume of 64.4 cm^3^, and material properties *c*_1_ = 2, *b*_ff_ = 4, *b*_xx_ = 2, *b*_fx_ = 8 in (19–20), and loaded with internal pressure 1 kPa. In the axisymmetric FE simulations, the two-dimensional domain initially contained 38 triangular elements. The mesh was refined to 183 and then 375 elements to test convergence. The current model and FE solutions are compared in Fig. 4. Displacements of the low-order model agree closely with the finite element solution with small deviations near the apex. Distributions of deviatoric stress components generally agree closely, with differences appearing most prominently in areas of high curvature. The nonlinear FE model required 5.6 h to solve and the current model required 4 s to reach an equilibrium solution on a Dell Latitude E6420 Intel Core i5-2520 M with 2.5 Ghz processor with 4 GB RAM (Dell Computer Corporation, Austin TX).

### Model Pressures, Volumes and Strains for Several Cardiac Cycles

**Figure 5:**
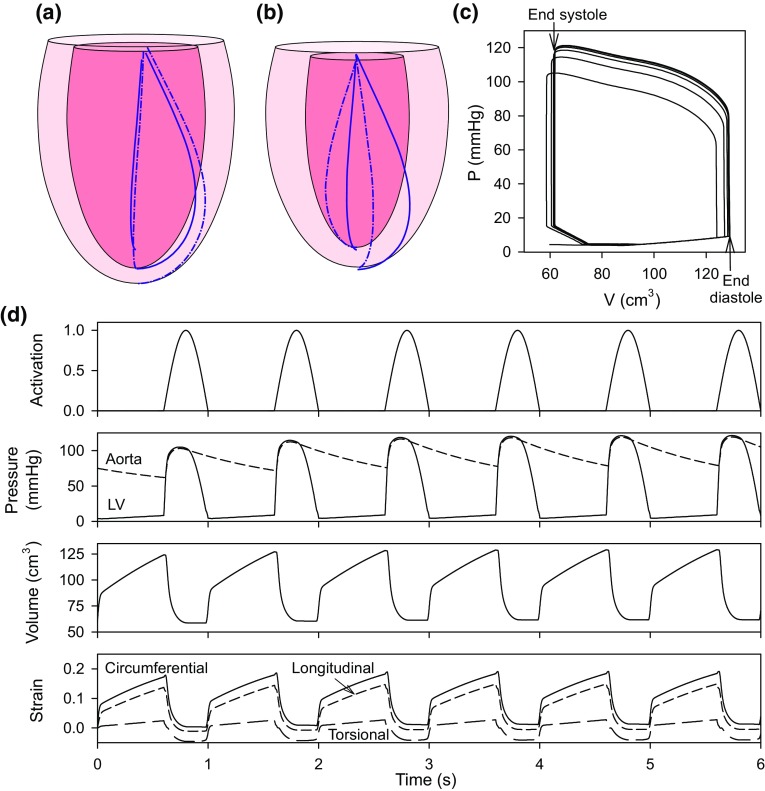
Examples of model results. (**a**) End diastolic configuration. (**b**) End systolic configuration. Reference endocardial and epicardial fiber curves are shown. LV torsion and twist of the fibers are demonstrated (dashed lines). (**c**) Pressure–volume curves for twelve cycles. (**d**). Time course of assumed activation, LV and aortic pressures, LV volume, and strain components over 6 cycles. The strain components *E*_*ϕϕ*_ (circumferential), *E*_*νν*_ (longitudinal) and *E*_*νϕ*_ (torsional) are computed at a point in the mid-wall where *ν*_0_ = 3π/4.

The model was solved over the period of twelve cardiac cycles, for the model with normal shape and parameters given in Table 1. Solution time was 7.1 s, *i.e.*, 0.6 s per cardiac cycle. End diastolic and end-systolic LV geometry are shown in Figs. 5a and 5b (for the twelfth cardiac cycle) along with reference fibers (solid lines) on the endocardium and epicardium superimposed on the deformed configurations (dashed lines). Corresponding pressure–volume curves are shown in Fig. 5c. Figure 5d shows the time course of activation, LV and aortic pressures, LV volume, and strain components over the first 6 cycles of the simulation. Periodic behavior is achieved after 4–5 cycles.

### Effects of LV Ellipticity on Pressure Generation and Wall Stresses

**Figure 6:**
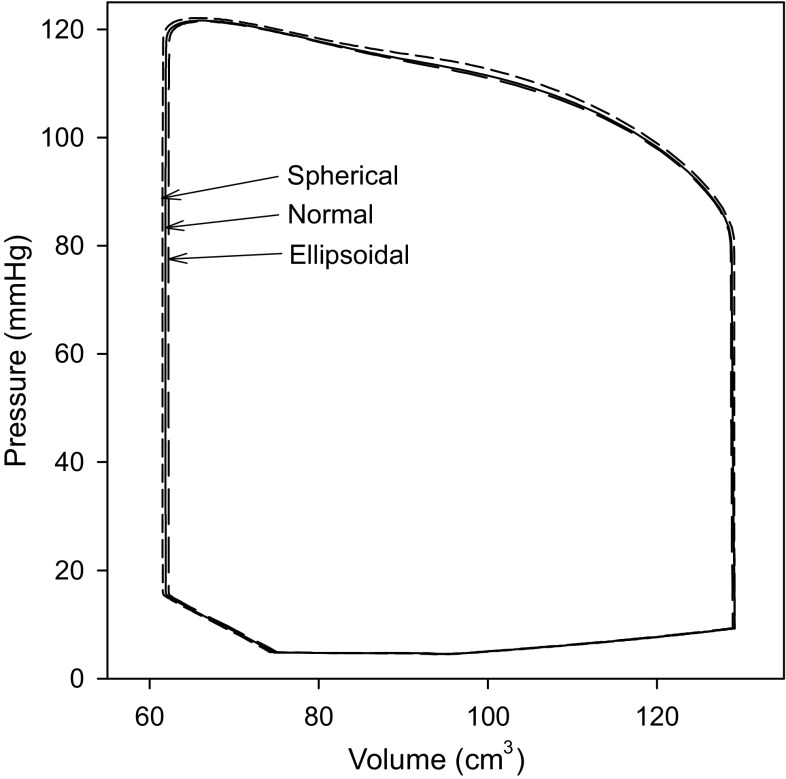
Pressure–volume loops for three different reference shapes with same wall and cavity volumes. Solid line: normal. Short dashed line: spherical. Long dashed line: ellipsoidal.

For each of the LV models with different shapes (spherical, normal, ellipsoidal) shown in Fig. 3, model solutions were obtained with the same passive and active material properties, viscous parameters and circulatory model parameters. Figure 6 shows the pressure–volume loops for the three shapes, which agree very closely, despite the different LV shapes. The stroke work values for the three models agree within about 1% (Table 1). Diastolic filling commences slightly before the end of muscle activation, resulting in a brief period during which pressure declines while volume is increasing.

By integrating over time, (25) can be used to calculate the work done during computed motions of the LV. For the normal geometry, the internal work done per cycle (averaged over twelve cardiac cycles) is 0.915 J/cycle, representing the sum of the fiber work (1.004 J/cycle) and the viscous dissipation in the myocardium (−0.089 J/cycle). The net change in elastic energy is zero over each cycle. The stroke work, computed as $\int {pdV}$, is 0.900 J/cycle. Corresponding results for the spherical and ellipsoidal geometries are given in Table 1. In theory, the net internal work should equal the stroke work. The differences found here (respectively 1.6, 0.4 and 4.7%) are attributed to the approximate estimate (S67) of the work done at the base of the heart. The analysis was repeated for three corresponding models with the same internal and wall volumes, but with *ν*_up_ = π/2, so that there is no *z* displacement at the base. In that case, stroke work and net internal work agreed within 0.001%.

**Figure 7:**
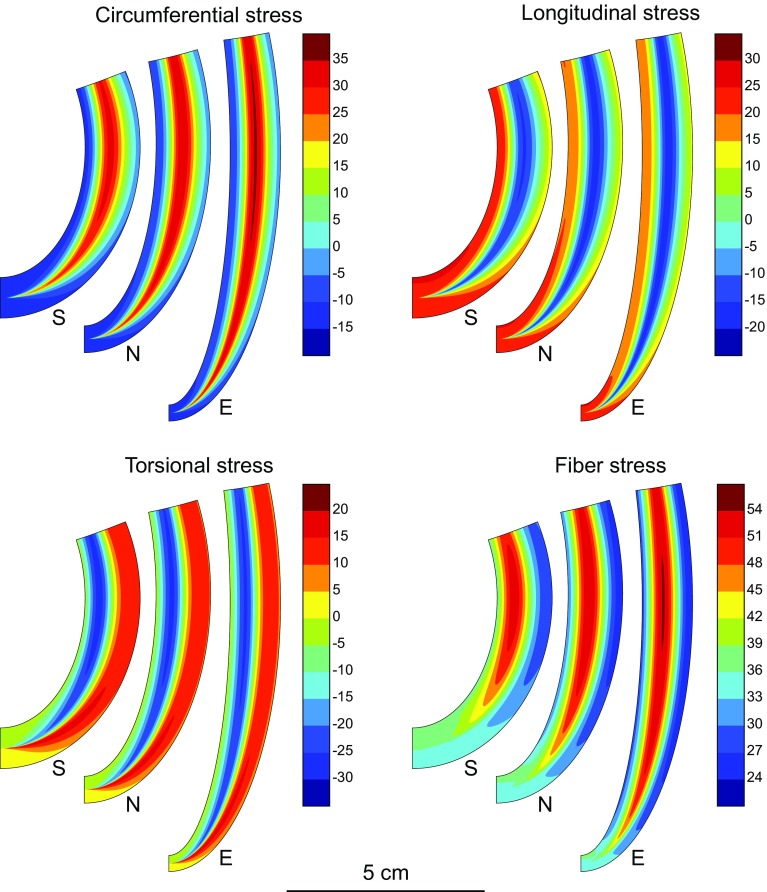
Deviatoric wall stress contours at peak systole for three different reference shapes with same wall and cavity volumes. For each stress component, shapes are (left to right) spherical (S), normal (N) and ellipsoidal (E). Color bar scales in kPa.

**Figure 8:**
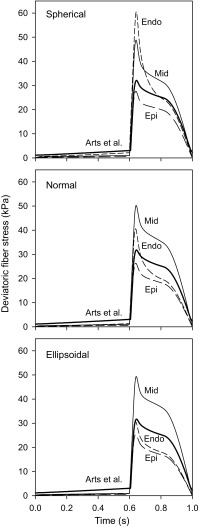
Variation of deviatoric normal stress in the fiber direction through the cardiac cycle, computed at points at the endocardium, mid-wall and epicardium where *ν*_0_ = 3π/4. Heavy curves show predictions of model by Arts *et al*.,^1^ based on uniform fiber stress throughout myocardium and the computed cavity pressures.

The deviatoric components of the Cauchy stresses *σ*_*ϕϕ*_, *σ*_*νν*_, *σ*_*ϕν*_ and *σ*_*ff*_ for the three shapes at peak activation are shown in Fig. 7. The spatial patterns and magnitudes of the peak systolic stresses are similar for all three LV shapes. The strong variations of circumferential and longitudinal stresses through the wall reflect the variations in fiber orientation. Fiber stress varies across the wall, with a maximum near the mid-wall, where fibers are nearly circumferential, near the LV equator. Towards the apex, fiber stresses are lower and the maximum occurs at the endocardium. Figure 8 shows the predicted time-dependent variation of deviatoric stress in the fiber direction at three locations through the wall (endocardium, midwall and epicardium) at *ν*_0_ = 3π/4, for the three LV shapes. The fiber stresses predicted by the model of Arts *et al*.^1^ are also shown, as computed using (34) with the cavity pressure from the current model.

### Sensitivity of Results to Reference Parameter Values and Cavity-to-Wall Volume Ratio

The predictions of these simulations were tested by varying passive muscle, active muscle and circulatory reference parameter values by ±20%. Close agreement of pressure–volume curves and stroke work and qualitative agreement of fiber stress distributions for the three geometries were found throughout the parameter range. The relationship between the Arts *et al*.^1^ model and the current results was found to be similar throughout the range of parameters examined, and also when models with cavity to wall volume ratios of 0.2 and 0.6 were examined.

## Discussion

The overall goal of this work is to develop a fast computational model that links the kinematics of the beating LV with the active and passive properties of the myocardial tissue. To address this goal, we developed a model with a small number of degrees of freedom. This approach can be considered as intermediate in level of detail between the simplicity of lumped approaches such as the varying elastance model and the complexity of finite-element approaches with high spatial resolution. As such, it combines an explicit description of LV geometry and deformation with low computational cost.

The present model has several distinctive features. (i) The passive properties of the myocardium are represented using a Kelvin–Voigt viscoelastic model, with elastic properties derived from a transversely isotropic strain-energy function previously used to model the heart.^13^ (ii) Nonlinear (finite deformation) kinematics and material properties are utilized. (iii) Active contraction is represented by a model of muscle that incorporates length–tension and force–velocity properties and where force generation depends on end-diastolic strain according to the Frank–Starling law. Muscle activation is a prescribed function of time, such as could be derived from an electrophysiological model. (iv) The LV is coupled to a lumped-parameter model of the circulatory system that includes opening and closing of cardiac valves. (v) The model predicts LV strains, volume, stresses and pressure throughout the cardiac cycle. (vi) Because it is a low-order model, it is executed faster than real time, with potential applications for on-line assessment of muscle properties using clinically available data including echocardiography.

The low-order model represents a compromise between physiological realism and computational speed. Only the LV is modeled, and effects of interactions with the right ventricle (RV),^8^ either by direct mechanical interaction or through interactions with the pulmonary circulation, are neglected. The LV is assumed to be axisymmetric, and effects that depend on angular position, such as LV-RV interactions or localized myocardial damage, are not included. Such effects could potentially be incorporated by adding additional degrees of freedom to the mapping functions. A phenomenological model for muscle contraction is used. Incorporation of a model for the biophysics of sarcomere function^16^,^22^ could yield more insight into the interplay between fiber contraction and relaxation and ventricular performance.

In the case of passive expansion of a pressurized spheroid, close agreement was found between the low-order model and finite element simulations with many degrees of freedom (Fig. 4). This finding suggests that a low-order approach can faithfully represent important aspects of LV mechanics.

The results in Fig. 5 illustrate the ability of the model to predict a range of parameters and behaviors that are of primary interest with regard to LV dynamics, including LV pressure and internal volume, components of stress and strain, P–V loops, and three-dimensional LV motions including contraction, shortening and torsion. This computation ran faster than real time and has potential to run much faster.

An important contribution to the understanding of the relationship between myocardial stress and LV pressure was made by Arts *et al*.^1^ Assuming that fiber stress is uniform throughout the wall, they derived Eq. (34) giving the ratio of LV pressure to fiber stress to as a function of the ratio of wall volume to cavity volume, for any axisymmetric shape and independent of fiber orientation. This equation implies that LV shape does not have a large effect on pressure generation for given fiber stress and given cavity and wall volumes. Our results are consistent with this implication. The pressure–volume loops for three LV models with matched volumes but different shapes (Fig. 3) agree closely (Fig. 6), with only 1% variation in stroke work between models (Table 1).

In contrast to the model of Arts *et al*.,^1^ the present model allows estimation of variations in fiber stress and strain through the LV wall. Figure 7 shows substantial spatial variations in fiber stress at peak systolic contraction. Figure 8 shows that the temporal variation of fiber stress, although similar in form to that predicted by the model of Arts *et al*., varies significantly through the wall. In the model of Arts *et al*., fiber stress is assumed to be uniform from endocardium to epicardium. Their model does not include passive stiffness, and does not allow prediction of diastolic stresses. Also, it does not explicitly describe torsional stresses or deformation. The results in Figs. 7 and 8 are based on an assumed reference configuration that was taken to be close to the end-systolic echo geometry. Initially, all sarcomere lengths are equal at the reference shape with value of 1.82 *μ*m. As the heart expands and contracts, the fiber strain is spatially variable, resulting in a non-uniform distribution of fiber stress. Even if a different reference shape was assumed, there would still be transmural variations in fiber strain and therefore variations of stress, in contrast to the Arts *et al*. model (Table 2).

**Table 2:** Parameters and variables of the model.

Parameter	Value	Description
$a,a_{1} ,a_{2} ,a_{3}$		Kinematic parameters defining deformation field
$b_{ff}$	5	Passive elastic material coefficient
$b_{fx}$	7	Passive elastic material coefficient
$b_{xx}$	3	Passive elastic material coefficient
$c_{1}$	1	Passive nonlinear material coefficient (kPa)
$C_{A}$	20	Compliance of atrium (cm^3^/kPa)
$C_{SYST}$	10	Compliance of aorta (cm^3^/kPa)
${\mathbf{F}}$		Deformation gradient tensor
$F_{0}$	300	Active force generation parameter (kPa)
$g_{\mu } ,g_{\nu } ,g_{\phi }$		Scale factors for ellipsoidal coordinates
${\mathbf{J}}$		Jacobian of mapping from prolate to Cartesian coordinates
$k$	0	Sensitivity of active force to end-diastolic fiber strain
$k^{\prime}$	1	Controls shape of activation function
$k_{av}$	0.5	Constant controlling amount of viscous fiber stress
$k_{vm}$	0.025	Constant reflecting the amount of viscous matrix stress
$L_{s0}$	1.82	Length of sarcomere at zero pressure (*μ*m)
$L_{s}$		Dynamic sarcomere length (*μ*m)
$L_{s\hbox{max} }$	2.23	Length of sarcomere (*μ*m) where maximum tension is generated
$L_{sw}$	0.2	Width of exponential length–tension relationship (*μ*m)
$p$		LV pressure (kPa), variable
$P_{A}$		Atrial pressure (kPa), variable
$P_{AO}$		Pressure in the proximal aorta (kPa), variable
$P_{AOV}$		Aortic pressure, just beyond aortic valve (kPa), variable
$P_{SV}$	6.0	Pressure in the systemic veins (kPa)
$P_{PA}$	3.0	Pressure in the pulmonary circulation (kPa)
$R_{PULM}$	0.023	Resistance in pulmonary circulation (kPa s/cm^3^)
$R_{MV}$		Resistance of mitral valve (open) (kPa s/cm^3^), variable
$R_{OP_MV}$	0.0008	Resistance of fully open mitral valve (kPa s/cm^3^)
$R_{AOV}$		Resistance of aortic valve (open) (kPa s/cm^3^), variable
$R_{OP_AOV}$	0.0005	Resistance of fully open aortic valve (kPa s/cm^3^)
$R_{AO}$	0.001	Proximal aortic resistance, constant (kPa s/cm^3^)
$R_{SYST}$	0.11	Resistance of systemic circulation (kPa s/cm^3^)
${\mathbf{S}},{\mathbf{S}}_{f} ,{\mathbf{S}}_{e} ,{\mathbf{S}}_{v}$		PK2 stress tensors: total, fiber, elastic, viscous
${\mathbf{T}}_{{\mathbf{b}}} ,{\mathbf{T}}_{{\mathbf{e}}}$		Boundary tractions: base of LV, endocardium
$\varOmega_{0} ,\;\Omega$		Domain of the LV myocardium: reference, deformed configuration
${\mathbf{E}}$		Green strain tensor
${\mathbf{u}}$		Boundary displacements for virtual work equations
$T_{a}$	0.4	Period of ventricular activation (s)
$T_{c}$	1	Period of cardiac cycle (s)
$\mu ,\mu_{0}$		Coordinate for position through wall: deformed, reference
$\nu_{0}$		Base-to-apex coordinate
$\phi ,\phi_{0}$		Coordinate for circumferential position: deformed, reference
$\psi$		Local fiber angle
$\psi^{*}$		Quadrant adjusted local fiber angle
$\omega$		Fiber wrapping parameter
$ds_{0} ,\;ds$		Fiber arc length: reference, deformed configuration

Ventricular wall stress is an important determinant of muscle metabolism and growth and remodeling in normal and pathologic conditions,^38^ but it is not measurable in the beating heart. Key aspects of muscle mechanics such as force–velocity and force–length relationships are measurable in isolated muscle specimens but not in the intact LV. The model developed here provides a basis for noninvasive assessment of wall stress and active force development in the LV. Predictions of the pattern and magnitude of longitudinal, circumferential, torsional and fiber stresses, as shown in Fig. 7, have potential applications for investigating the mechanisms of normal and pathological remodeling of the myocardium, and for estimating the spatial distribution (transmural and base to apex) of metabolic needs in the LV.

A goal of this work is to utilize observed LV deformations from speckle tracking echocardiography, in conjunction with the dynamic model, to identify parameters that define active and passive muscle properties. Such parameter identification will require repeated simulations of LV motion and deformation using the model, with iterative adjustment of the parameters for best fit between predicted and observed motions. The rapid computational speed of the present model makes this approach feasible. Alternatively, low-order model parameter estimation could serve as a preliminary step for finite-element model parameter estimation, narrowing the parameter space and thereby reducing computational needs. The model presented here assumes axisymmetric LV geometry, but the low-order approach can be extended to the analysis of non-axisymmetric geometries by including additional modes of deformation.

## Electronic supplementary material

Below is the link to the electronic supplementary material.

Supplementary material 1 (DOCX 287 kb)
