# Supplementary material for: Simulation of Left Ventricular Dynamics Using a Low-Order Mathematical Model
Source: Cardiovasc Eng Technol. 2017 Aug 15;8(4):480–94. doi: 10.1007/s13239-017-0327-9 (PMC5707240; doi:10.1007/s13239-017-0327-9)
Supplement: Supplementary file 1 — Supplementary material 1 (DOCX 287 kb) [file 13239_2017_327_MOESM1_ESM.docx]

**Supplementary Material**

**Simulation of Left Ventricular Dynamics Using a Low-Order Mathematical Model**

Michael J. Moulton^1^, Brian D. Hong^2^ and Timothy W. Secomb^3^

**Deformation gradient tensor and Green-Lagrange strain tensor**

From equations (1-6), the deformation gradient tensor in prolate spheroidal coordinates is:

$\mathbf{F}=\left[\begin{matrix} F_{\mu\mu} & F_{\mu\nu} & 0 \\ 0 & F_{\nu\nu} & 0 \\ 0 & F_{\varphi\nu} & F_{\varphi\varphi} \end{matrix}\right]$ (S1)

The components are

$F_{\mu\mu}=\frac{g_{\mu}}{g_{\mu0}}\frac{\partial\mu}{\partial\mu_{0}}=\frac{a\sqrt{\sinh^{2}\mu+\sin^{2}\nu_{0}}}{a_{0}\sqrt{\sinh^{2}\mu_{0}+\sin^{2}\nu_{0}}}\frac{\partial\mu}{\partial\mu_{0}}$ (S2)

$F_{\mu\nu}=\frac{g_{\mu}}{g_{\nu0}}\frac{\partial\mu}{\partial\nu_{0}}=\frac{a\sqrt{\sinh^{2}\mu+\sin^{2}\nu_{0}}}{a_{0}\sqrt{\sinh^{2}\mu_{0}+\sin^{2}\nu_{0}}}\frac{\partial\mu}{\partial\nu_{0}}$ (S3)

$F_{\nu\nu}=\frac{g_{\nu}}{g_{\nu0}}\frac{\partial\nu}{\partial\nu_{0}}=\frac{a\sqrt{\sinh^{2}\mu+\sin^{2}\nu_{0}}}{a_{0}\sqrt{\sinh^{2}\mu_{0}+\sin^{2}\nu_{0}}}$ (S4)

$F_{\varphi\nu}=\frac{g_{\varphi}}{g_{\nu0}}\frac{\partial\varphi}{\partial\nu_{0}}=-\frac{a_{3}a\sinh\mu\sin^{2}\nu_{0}}{a_{0}\sqrt{\sinh^{2}\mu_{0}+\sin^{2}\nu_{0}}}$ (S5)

$F_{\varphi\varphi}=\frac{g_{\varphi}}{g_{\varphi0}}\frac{\partial\varphi}{\partial\varphi_{0}}=\frac{a\sinh\mu}{a_{0}\sinh\mu_{0}}$ (S6)

where

$\frac{\partial\mu}{\partial\nu_{0}}=\frac{2\cos\nu_{0}\sin\nu_{0}\left[-a^{3}(\cosh\mu-1)+a_{0}^{3}(\cosh\mu_{0}-1)-a_{2}a_{0}^{2}(\cosh\mu_{in0}-1)\right]}{a^{3}\sinh\mu(\sinh^{2}\mu+\sin^{2}\nu_{0})}$ (S7)

$\frac{\partial\mu}{\partial\mu_{0}}=\frac{a_{0}^{3}\sinh\mu_{0}(\sinh^{2}\mu_{0}+\sin^{2}\nu_{0})}{a^{3}\sinh\mu(\sinh^{2}\mu+\sin{}^{2}\nu_{0})}$ (S8)

and so

$F_{\mu\mu}=\frac{a_{0}^{2}\sinh\mu_{0}\sqrt{\sinh^{2}\mu_{0}+\sin^{2}\nu_{0}}}{a^{2}\sinh\mu\sqrt{\sinh^{2}\mu+\sin^{2}\nu_{0}}}$ (S9)

and $\det(\mathbf{F})=F_{\mu\mu}F_{\nu\nu}F_{\varphi\varphi}=1$ as expected. The components of the Green-Lagrange strain tensor are:

(S10)

(S11)

(S12)

(S13)

(S14)

$E_{\mu\varphi}=0$ (S15)

**Base vectors in prolate spheroidal and fiber coordinate systems**

In the reference configuration, the base vectors $(\mathbf{e}_{\mu},\mathbf{e}_{\nu},\mathbf{e}_{\varphi})$ of the prolate spheroidal coordinate system (where *φ* = 0 without loss of generality) are:

$\mathbf{e}_{\mu}=\frac{\cosh\mu_{0}\sin\nu_{0}\mathbf{e}_{x}+\sinh\mu_{0}\cos\nu_{0}\mathbf{e}_{z} }{\sqrt{\sinh^{2}\mu_{0}+\sin^{2}\nu_{0}}}$ (S16) $\mathbf{e}_{\nu}=\frac{\sinh\mu_{0}\cos\nu_{0}\mathbf{e}_{x}-\cosh\mu_{0}\sin\nu_{0}\mathbf{e}_{z}}{\sqrt{\sinh^{2}\mu_{0}+\sin^{2}\nu_{0}}}$ (S17)

$\mathbf{e}_{\varphi}=\mathbf{e}_{y}$ (S18)

The fiber paths in the reference configuration are expressed parametrically by

$\begin{matrix} x\left(\mu_{0},\varphi_{b};\nu_{0}\right)=a_{0}\sinh\mu_{0}\sin\nu_{0}\cos[\varphi_{b}+\omega(\cos\nu_{0}-\cos\nu_{up})] \\ y\left(\mu_{0},\varphi_{b};\nu_{0}\right)=a_{0}\sinh\mu_{0}\sin\nu_{0}\sin[\varphi_{b}+\omega(\cos\nu_{0}-\cos\nu_{up})] \\ z\left(\mu_{0};\nu_{0}\right)=a_{0}\cosh\mu_{0}\cos\nu_{0} \end{matrix}$ (S19)

The base vectors $(\mathbf{e}_{s},\mathbf{e}_{n},\mathbf{e}_{f})$ of the fiber coordinate system (where *φ* = 0) are:

$\mathbf{e}_{s}=\mathbf{e}_{\mu}$ (S20)

$\mathbf{e}_{n}=\mathbf{e}_{f}\times\mathbf{e}_{s}$ (S21)

$\mathbf{e}_{f}=\frac{\sinh\mu_{0}\cos\nu_{0}\mathbf{e}_{x}-\omega\sinh\mu_{0}\sin^{2}\nu_{0}\mathbf{e}_{y}-\cosh\mu_{0}\sin\nu_{0}\mathbf{e}_{z}}{\sqrt{\sinh^{2}\mu_{0}+\sin^{2}\nu_{0}+\omega^{2}\sinh^{2}\mu_{0}\sin^{4}\nu_{0} }}$ (S22)

The angle $\psi^{*}$ is defined as the angle that the negative fiber direction $-\mathbf{e}_{f}$ makes with the $\varphi$-coordinate direction $\mathbf{e}_{\varphi}$ in the reference configuration as shown in Figure 1C. Therefore

$\cos\psi^{*}=(-\mathbf{e}_{f})\cdot\mathbf{e}_{\varphi}=\frac{\omega\sinh\mu_{0}\sin^{2}\nu_{0}}{\sqrt{\sinh^{2}\mu_{0}+\sin^{2}\nu_{0}+\omega^{2}\sinh^{2}\mu_{0}\sin^{4}\nu_{0}}}$ (S23)

$\sin\psi^{*}=\mathbf{e}_{f}\cdot\mathbf{e}_{\nu}=\frac{\sqrt{\sinh^{2}\mu_{0}+\sin^{2}\nu_{0}}}{\sqrt{\sinh^{2}\mu_{0}+\sin^{2}\nu_{0}+\omega^{2}\sinh^{2}\mu_{0}\sin^{4}\nu_{0}}}$ (S24)

Using the definition of the base vectors, a simple rotation matrix can be derived to convert vectors and tensors from the fiber Cartesian system to the prolate direction:

$\mathbf{Q}=\left[\begin{matrix} \mathbf{e}_{s}\cdot\mathbf{e}_{\mu} & \mathbf{e}_{n}\cdot\mathbf{e}_{\mu} & \mathbf{e}_{f}\cdot\mathbf{e}_{\mu} \\ \mathbf{e}_{s}\cdot\mathbf{e}_{\nu} & \mathbf{e}_{n}\cdot\mathbf{e}_{\nu} & \mathbf{e}_{f}\cdot\mathbf{e}_{\nu} \\ \mathbf{e}_{s}\cdot\mathbf{e}_{\varphi} & \mathbf{e}_{n}\cdot\mathbf{e}_{\varphi} & \mathbf{e}_{f}\cdot\mathbf{e}_{\varphi} \end{matrix}\right]=\left[\begin{matrix} 1 & 0 & 0 \\ 0 & -\cos\psi^{*} & \sin\psi^{*} \\ 0 & -\sin\psi^{*} & -\cos\psi^{*} \end{matrix}\right]$ (S25)

To compute the work integrals (see below), the components of the stress tensors are converted from fiber coordinates to the undeformed prolate coordinates $\left(\mu_{0},\nu_{0},\varphi_{0}\right)$. The components of a PK2 stress in prolate coordinates are related to the components in fiber coordinates by

$\mathbf{S}^{\mu\nu\varphi}=\mathbf{QS}^{snf}\mathbf{Q}^{T}$ (S26)

**Derivation of viscous stress equation**

Following eq. (28), the viscous stress of the matrix $\sigma_{v}$is assumed to be additive to the elastic stress and further assumed to take the form of that in a viscous fluid. In the current configuration:

(S27)

where $U_{i}=\frac{\partial x_{i}}{\partial t}$in Cartesian coordinates. Then we let

$x_{i}=x_{i}\left(X_{j},t)\right), U_{i}=\frac{\partial x_{i}}{\partial t}, \frac{\partial U_{i}}{\partial x_{j}}=\frac{\partial u_{i}}{\partial X_{k}}\frac{\partial X_{k}}{\partial x_{j}}=\frac{\partial^{2}x_{i}}{\partial X_{k}\partial t}\frac{\partial X_{k}}{\partial x_{j}}$ (S28)

giving

. (S29)

The second Piola-Kirchhoff stress satisfies

(S30)

where $\mathbf{f}=\mathbf{C}^{-\mathbf{1}}$is the finger strain and $\mathbf{C}=\frac{\partial x_{k}}{\partial X_{i}}\frac{\partial x_{k}}{\partial X_{j}}$and $f_{ij}=\frac{\partial X_{i}}{\partial x_{k}}\frac{\partial X_{j}}{\partial x_{k}}$. Therefore,

$\begin{matrix} S_{v mn}=k_{vm}J f_{mr}f_{nk}\left(\frac{\partial x_{i}}{\partial X_{r}}\frac{\partial^{2}x_{i}}{\partial X_{k}\partial t}+\frac{\partial x_{i}}{\partial X_{k}}\frac{\partial^{2}x_{i}}{\partial X_{r}\partial t}\right) \\ =k_{vm}J f_{mr}f_{nk}\frac{\partial}{\partial t} \left(\frac{\partial x_{i}}{\partial X_{r}}\frac{\partial x_{i}}{\partial X_{k}}\right) \end{matrix}$ (S31)

In this case, *J* = 1. On substituting , the Cauchy rate of deformation tensor, we have, since $\mathbf{f}$is symmetric,

. (S32)

**Derivatives of the Green-Lagrange strain tensor**

The derivatives of these quantities with respect to the kinematic parameters *a*_1_, *a*_2_ and *a*_3_ are needed, where *a* = *a*_0_ + *a*_1_. Then:

$\begin{matrix} \frac{\partial E_{\mu\mu}}{\partial a_{1}}=-\frac{a_{o}^{4}\sinh^{2}\mu_{0}(\sinh^{2}\mu_{0}+\sin^{2}\nu_{0})}{a^{5}\sinh^{3}\mu(\sinh^{2}\mu+\sin^{2}\nu_{0})^{2}} \\ \cdot\left(2\sinh\mu(\sinh^{2}\mu+\sin^{2}\nu_{0})+a\cosh\mu(2\sinh^{2}\mu+\sin^{2}\nu_{0})\frac{\partial\mu}{\partial a_{1}}\right) \end{matrix}$ (S33)

$\frac{\partial E_{\mu\mu}}{\partial a_{2}}=-\frac{a_{o}^{4}\sinh^{2}\mu_{0}(\sinh^{2}\mu_{0}+\sin^{2}\nu_{0})}{a^{4}\sinh^{3}\mu(\sinh^{2}\mu+\sin^{2}\nu_{0})^{2}}\cosh\mu(2\sinh^{2}\mu+\sin^{2}\nu_{0})\frac{\partial\mu}{\partial a_{2}}$ (S34)

$\frac{\partial E_{\mu\mu}}{\partial a_{3}}=0$ (S35)

$\begin{matrix} \frac{\partial E_{\nu\nu}}{\partial a_{1}}=\frac{a^{2}(\sinh^{2}\mu+\sin^{2}\nu_{0})}{a_{0}^{2}(\sinh^{2}\mu_{0}+\sin^{2}\nu_{0})}\frac{\partial\mu}{\partial\nu_{0}}\frac{\partial}{\partial a_{1}}\left(\frac{\partial\mu}{\partial\nu_{0}}\right) \\ +\left(1+\left(\frac{\partial\mu}{\partial\nu_{0}}\right)^{2}\right)\left(\frac{a(\sinh^{2}\mu+\sin^{2}\nu_{0})}{a_{0}^{2}(\sinh^{2}\mu_{0}+\sin^{2}\nu_{0})}+\frac{a^{2}\sinh\mu\cosh\mu}{a_{0}^{2}(\sinh^{2}\mu_{0}+\sin^{2}\nu_{0})}\frac{\partial\mu}{\partial a_{1}}\right) \\ +\frac{a_{3}^{2}a\sinh\mu\sin^{4}\nu_{0}}{a_{0}^{2}(\sinh^{2}\mu_{0}+\sin^{2}\nu_{0})}\left(\sinh\mu+a\cosh\mu\frac{\partial\mu}{\partial a_{1}}\right) \end{matrix}$ (S36) $\begin{matrix} \frac{\partial E_{\nu\nu}}{\partial a_{2}}=\frac{a^{2}(\sinh^{2}\mu+\sin^{2}\nu_{0})}{a_{0}^{2}(\sinh^{2}\mu_{0}+\sin^{2}\nu_{0})}\frac{\partial\mu}{\partial\nu_{0}}\frac{\partial}{\partial a_{2}}\left(\frac{\partial\mu}{\partial\nu_{0}}\right)+\frac{a_{3}^{2}a^{2}\sinh\mu\cosh\mu\sin^{4}\nu_{0}}{a_{0}^{2}(\sinh^{2}\mu_{0}+\sin^{2}\nu_{0})}\frac{\partial\mu}{\partial a_{2}} \\ +\left(1+\left(\frac{\partial\mu}{\partial\nu_{0}}\right)^{2}\right)\frac{a^{2}\sinh\mu\cosh\mu}{a_{0}^{2}(\sinh^{2}\mu_{0}+\sin^{2}\nu_{0})}\frac{\partial\mu}{\partial a_{2}} \end{matrix}$ (S37)

$\frac{\partial E_{\nu\nu}}{\partial a_{3}}=\frac{a_{3}a^{2}\sinh^{2}\mu\sin^{4}\nu_{0}}{a_{0}^{2}(\sinh^{2}\mu_{0}+\sin^{2}\nu_{0})}$ (S38)

$\frac{\partial E_{\varphi\varphi}}{\partial a_{1}}=\frac{a\sinh\mu}{a_{0}^{2}\sinh^{2}\mu_{0}}\left(\sinh\mu+a\cosh\mu\frac{\partial\mu}{\partial a_{1}}\right)$ (S39)

$\frac{\partial E_{\varphi\varphi}}{\partial a_{2}}=\frac{a^{2}\sinh\mu\cosh\mu}{a_{0}^{2}\sinh^{2}\mu_{0}}\frac{\partial\mu}{\partial a_{2}}$ (S40)

$\frac{\partial E_{\varphi\varphi}}{\partial a_{3}}=0$ (S41) $\frac{\partial E_{\mu\nu}}{\partial a_{1}}=\frac{a_{0}\sinh\mu_{0}}{2a\sinh\mu}\left[\frac{\partial}{\partial a_{1}}\left(\frac{\partial\mu}{\partial\nu_{0}}\right)-\left(\frac{1}{a}+\frac{\cosh\mu}{\sinh\mu}\frac{\partial\mu}{\partial a_{1}}\right)\frac{\partial\mu}{\partial\nu_{0}}\right]$ (S42)

$\frac{\partial E_{\mu\nu}}{\partial a_{2}}=\frac{a_{0}\sinh\mu_{0}}{2a\sinh\mu}\frac{\partial}{\partial a_{2}}\left(\frac{\partial\mu}{\partial\nu_{0}}\right)-\frac{a_{0}\sinh\mu_{0}\cosh\mu}{2a\sinh^{2}\mu}\frac{\partial\mu}{\partial a_{2}}\frac{\partial\mu}{\partial\nu_{0}}$ (S43)

$\frac{\partial E_{\mu\nu}}{\partial a_{3}}=0$ (S44)

$\frac{\partial E_{\nu\varphi}}{\partial a_{1}}=-\frac{a_{3}a\sinh\mu\sin^{2}\nu_{0}}{a_{0}^{2}\sinh\mu_{0}\sqrt{\sinh^{2}\mu_{0}+\sin^{2}\nu_{0}}}\left(\sinh\mu+a\cosh\mu\frac{\partial\mu}{\partial a_{1}}\right)$ (S45)

$\frac{\partial E_{\nu\varphi}}{\partial a_{2}}=-\frac{a_{3}a^{2}\sinh\mu\cosh\mu\sin^{2}\nu_{0}}{a_{0}^{2}\sinh\mu_{0}\sqrt{\sinh^{2}\mu_{0}+\sin^{2}\nu_{0}}}\frac{\partial\mu}{\partial a_{2}}$ (S46)

$\frac{\partial E_{v\varphi}}{\partial a_{3}}=-\frac{a^{2}\sinh^{2}\mu\sin^{2}\nu_{0}}{2a_{0}^{2}\sinh\mu_{0}\sqrt{\sinh^{2}\mu_{0}+\sin^{2}\nu_{0}}}$ (S47)

From equation (5),

$\frac{\partial\mu}{\partial a_{1}}=\frac{-\cosh^{3}\mu+3\cosh\mu\cos^{2}\nu_{0}+1-3\cos^{2}\nu_{0}}{a\sinh\mu(\sinh^{2}\mu+\sin^{2}\nu_{0})}$ (S48)

$\frac{\partial\mu}{\partial a_{2}}=\frac{a_{0}^{2}\left(-\cosh^{3}\mu_{in0}+3\cosh\mu_{in0}\cos^{2}\nu_{0}+1-3\cos^{2}\nu_{0}\right)}{3a^{3}\sinh\mu(\sinh^{2}\mu+\sin^{2}\nu_{0})}$ (S49)

Also, we can write $\frac{\partial\mu}{\partial\nu_{0}}=\frac{f}{g}$ where

$f=2\cos\nu_{0}\sin\nu_{0}[a^{3}\left(1-\cosh\mu\right)+a_{0}^{3}\left(\cosh\mu_{0}-1\right)-a_{2}a_{0}^{2}\left(\cosh\mu_{in0}-1\right)]$ (S50) $g=a^{3}(\sinh^{3}\mu+\sinh\mu\sin^{2}\nu_{0})$ (S51)

Then $\frac{\partial}{\partial a_{1}}\left(\frac{\partial\mu}{\partial\nu_{0}}\right)$ and $\frac{\partial}{\partial a_{2}}\left(\frac{\partial\mu}{\partial\nu_{0}}\right)$ can be evaluated using the quotient rule with

$\frac{\partial f}{\partial a_{1}}=2\cos\nu_{0}\sin\nu_{0}\left[3a^{2}\left(1-\cosh\mu\right)-a^{3}\sinh\mu\frac{\partial\mu}{\partial a_{1}}\right]$ (S52)

$\frac{\partial g}{\partial a_{1}}=3a^{2}\left(\sinh^{3}\mu+\sinh\mu\sin^{2}\nu_{0}\right)+a^{3}\cosh\mu(3\sinh^{2}\mu+\sin^{2}\nu_{0})\frac{\partial\mu}{\partial a_{1}}$ (S53)

$\frac{\partial f}{\partial a_{2}}=-2\cos\nu_{0}\sin\nu_{0}\left(\cosh\mu_{in0}-1\right)\left[a^{3}\sinh\mu\frac{\partial\mu}{\partial a_{2}}+a_{0}^{2}\left(\cosh\mu_{in0}-1\right)\right]$ (S54)

$\frac{\partial g}{\partial a_{2}}=a^{3}\cosh\mu(3\sinh^{2}\mu+\sin^{2}\nu_{0})\frac{\partial\mu}{\partial a_{2}}$ (S55)

**Derivatives of the displacement vectors on the endocardial boundaries** $d\Omega_{e}$ **and** $\partial\Omega_{b}$

The displacement vector in the plane *φ* = 0 is

$\mathbf{u}=\left[\begin{matrix} u_{x} \\ u_{z} \end{matrix}\right]=\left[\begin{matrix} a\sinh\mu\sin\nu_{0}-a_{0}\sinh\mu_{0}\sin\nu_{0} \\ a\cosh\mu\cos\nu_{0}-a_{0}\cosh\mu_{0}\cos\nu_{0} \end{matrix}\right]$ (S56)

The displacement derivatives $\partial\mathbf{u}/\partial a_{i}$ are given by $\begin{matrix} \frac{\partial u_{x}}{\partial a_{1}}=\sinh\mu\sin\nu_{0}+a\cosh\mu\sin\nu_{0}\frac{\partial\mu}{\partial a_{1}} \\ \frac{\partial u_{z}}{\partial a_{1}}=\cosh\mu\cos\nu_{0}+a\sinh\mu\cos\nu_{0}\frac{\partial\mu}{\partial a_{1}} \\ \frac{\partial u_{x}}{\partial a_{2}}=a\cosh\mu\sin\nu_{0}\frac{\partial\mu}{\partial a_{2}} \\ \frac{\partial u_{z}}{\partial a_{2}}=a\sinh\mu\cos\nu_{0}\frac{\partial\mu}{\partial a_{2}} \end{matrix}$ (S57)

where $\partial\mu/\partial a_{1}$ and $\partial\mu/\partial a_{2}$ are defined in (S48) and (S49).

**Virtual work on the endocardial boundary** $d\Omega_{e}$

The unit vector normal to the inner boundary directed into the cavity in the plane *φ* = 0 has components

$(n_{x},n_{z})=\left(\frac{\partial z}{\partial\nu_{0}},-\frac{\partial x}{\partial\nu_{0}}\right)\left(\frac{\partial s}{\partial\nu_{0}}\right)^{-1}$ (S58)

where *s* is arc length along the inner boundary and from (1):

$\frac{\partial x}{\partial\nu_{0}}=a\sinh\mu\cos\nu_{0}+a\cosh\mu\sin\nu_{0}\frac{d\mu}{d\nu_{0}}$ (S59)

$\frac{\partial z}{\partial\nu_{0}}=-a\cosh\mu\sin\nu_{0}+a\sinh\mu\cos\nu_{0}\frac{d\mu}{d\nu_{0}}$ (S60)

$\left(\frac{\partial s}{\partial\nu_{0}}\right)^{2}=\left(\frac{\partial x}{\partial\nu_{0}}\right)^{2}+\left(\frac{\partial z}{\partial\nu_{0}}\right)^{2}=a^{2}\left(\sinh^{2}\mu+\sin^{2}\nu_{0}\right)\left(1+\left(\frac{d\mu}{d\nu_{0}}\right)^{2}\right)$ (S61)

The traction vector is given by

$(T_{x},T_{z})=-p(n_{x},n_{z})$ (S62)

The increment of area is

$dS=a\sinh\mu\sin\nu_{0}dsd\varphi=a\sinh\mu\sin\nu_{0} (\partial s/\partial\nu_{0})d\nu_{0}d\varphi$ (S63)

Therefore, the term in equation (25) defining the external virtual work on the surface $d\Omega_{e}$is

$\iint_{\partial\Omega_{e}} \frac{\partial\mathbf{u}}{\partial a_{i}}\cdot\mathbf{T}dS=-p\iint_{\partial\Omega_{e}} \left(\frac{\partial u_{x}}{\partial a_{i}}n_{x}+\frac{\partial u_{z}}{\partial a_{i}}n_{z}\right)dS=p\eta_{ei}$ (S64)

where

$\eta_{ei}=2\pi a\int_{\nu_{up}}^{\pi} \left(-\frac{\partial u_{x}}{\partial a_{i}}\frac{dz}{d\nu_{0}}+\frac{\partial u_{z}}{\partial a_{i}}\frac{\partial x}{\partial\nu_{0}}\right)\sinh\mu\sin\nu_{0} d\nu_{0}$ (S65)

for *i* = 1,2. There is no contribution for *i* = 3 since the derivatives with respect to$a_{3}$are zero.

**Virtual work on the myocardial boundary $\partial\Omega_{b}$ at the base of the heart**

The mappings defined by (5-6) generally imply a slight motion in the z-direction by the base of the heart (defined by *ν*_0_  = *ν_up_*), except in the special case ν_up_  = π/2. To compute this virtual work, we note that a shift of the entire LV in the z-direction as a rigid body must do zero virtual work. We consider that the LV cavity is bounded by the surface $z_{up}=a\cosh\mu_{in}\cos\nu_{up}$, corresponding to the upper edge of the endocardium (Figure S1). Because the pressure outside the epicardial surface of the LV is assumed to be zero, the virtual work done on the myocardial boundary $\partial\Omega_{b}$ is equal and opposite to that done on the upper disk of the cavity. It follows that


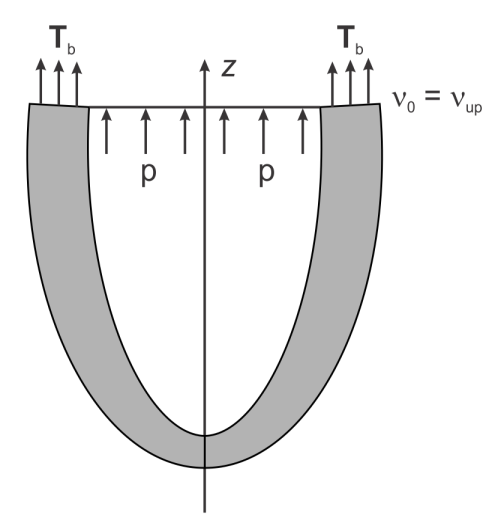


Figure S1. Virtual work at the base of the heart.

$\iint_{\partial\Omega_{b}} \frac{\partial\mathbf{u}_{b}}{\partial a_{i}}\cdot\mathbf{T}_{b}dS_{b}=p\eta_{bi}$ (S66)

where

$\eta_{bi}=\pi r_{up}^{2}\left.\frac{\partial u_{z}}{\partial a_{i}}\right|_{z_{up}}$ (S67)

and $r_{up}=a\sinh\mu_{in}\sin\nu_{up}$. The combined load integrals representing virtual work done on the boundaries are given by $\eta_{i}=\eta_{ei}+\eta_{bi}$.

**Virtual work equations**

The viscous stress can be rewritten in terms of the time derivatives of $a_{i}$:

(S68)

where

$\mathbf{S}_{vi}=k_{vm}\mathbf{C}^{-1}\frac{\partial\mathbf{C}}{\partial a_{i}}\mathbf{C}^{-1}$ (S69)

A similar manipulation of the fiber stress gives

$\begin{matrix} \mathbf{S}_{f}=\lambda_{f}^{-2}A\left(t\right)\left(F_{0}+k_{av}d\varepsilon_{f}/dt\right)(1+m\varepsilon_{f})(1+k\varepsilon_{f,ed})\mathbf{e}_{f}\otimes\mathbf{e}_{f} \\ =\mathbf{S}_{f0}+\lambda_{f}^{-2}(\mathbf{e}_{f}\otimes\mathbf{e}_{f})k_{av}A\left(t\right)\left(\frac{\partial\varepsilon_{f}}{\partial a_{1}}\frac{da_{1}}{dt}+\frac{\partial\varepsilon_{f}}{\partial a_{2}}\frac{da_{2}}{dt}+\frac{\partial\varepsilon_{f}}{\partial a_{3}}\frac{da_{3}}{dt}\right)(1+m\varepsilon_{f})(1+k\varepsilon_{f,ed}) \\ =\mathbf{S}_{f0}+\mathbf{S}_{f1}\frac{da_{1}}{dt}+\mathbf{S}_{f2}\frac{da_{2}}{dt}+\mathbf{S}_{f3}\frac{da_{3}}{dt} \end{matrix}$ (S70)

where

$\mathbf{S}_{f0}=\lambda_{f}^{-2}F_{0}A\left(t\right)(1+m\varepsilon_{f})(1+k\varepsilon_{f,ed})(\mathbf{e}_{f}\otimes\mathbf{e}_{f})$ (S71)
 $\mathbf{S}_{fi}=\lambda_{f}^{-2}k_{av}A\left(t\right)(1+m\varepsilon_{f})(1+k\varepsilon_{f,ed})(\mathbf{e}_{f}\otimes\mathbf{e}_{f})\frac{\partial\varepsilon_{f}}{\partial a_{i}}$ for $i=1,2,3$ (S72)

The force balance equations (26) can be restated as a system of differential equations for *a_i_* where the coefficients are the incremental virtual work integrals. The virtual work integrals are given in terms of the PK2 stresses (where each of the stress tensors is in the prolate spheroid coordinate system) as

$\alpha_{i}=\iiint\left(\frac{\partial\mathbf{E}}{\partial a_{i}}:\left(\mathbf{S}_{v1}+\mathbf{S}_{f1}\right)\right)dV_{0}$ $\beta_{i}=\iiint\left(\frac{\partial\mathbf{E}}{\partial a_{i}}:\left(\mathbf{S}_{v2}+\mathbf{S}_{f2}\right)\right)dV_{0}$ $\gamma_{i}=\iiint\left(\frac{\partial\mathbf{E}}{\partial a_{i}}:\left(\mathbf{S}_{v3}+\mathbf{S}_{f3}\right)\right)dV_{0}$ $\kappa_{i}=\iiint\left(\frac{\partial\mathbf{E}}{\partial a_{i}}:\left(\mathbf{S}_{e}+\mathbf{S}_{f0}\right)\right)dV_{0}$ (S73)

The load integrals are given by

$\eta_{i}=2\pi a\int_{\nu_{up}}^{\pi} \left(-\frac{\partial u_{x}}{\partial a_{i}}\frac{\partial z}{\partial\nu_{0}}+\frac{\partial u_{z}}{\partial a_{i}}\frac{\partial x}{\partial\nu_{0}}\right)\sinh\mu\sin\nu_{0} d\nu_{0}+\pi r_{up}^{2}\left.\frac{\partial u_{z}}{\partial a_{i}}\right|_{z_{up}}$ (S74)

Using these definitions the force balance equations (26) can be written as a system of three differential equations

$\left\{\begin{matrix} \alpha_{1}\frac{da_{1}}{dt}+\beta_{1}\frac{da_{2}}{dt}+\gamma_{1}\frac{da_{3}}{dt}+\kappa_{1}-\eta_{1}p=0 \\ \alpha_{2}\frac{da_{1}}{dt}+\beta_{2}\frac{da_{2}}{dt}+\gamma_{2}\frac{da_{3}}{dt}+\kappa_{2}-\eta_{2}p=0 \\ \alpha_{3}\frac{\partial a_{1}}{dt}+\beta_{3}\frac{da_{2}}{dt}+\gamma_{3}\frac{da_{3}}{dt}+\kappa_{3}=0 \end{matrix}\right.$ (S75)

**Rate of change of volume**

The ventricular volume is computed as a solid of revolution from the inner wall curve in the *φ* = 0 plane to be

$\begin{matrix} V=\pi\int_{z_{0}}^{z_{1}} x^{2}dz=-\pi a^{2}\int_{\pi}^{\nu_{up}} \sinh^{2}\mu\sin^{2}\nu_{0}\frac{\partial z}{\partial\nu_{0}}d\nu_{0} \\ =\pi a^{3}\int_{\nu_{up}}^{\pi} \sinh^{2}\mu\sin^{2}\nu_{0}\left(\cosh\mu\sin\nu_{0}-\sinh\mu\cos\nu_{0}\frac{\partial\mu}{\partial\nu_{0}}\right)d\nu_{0} \end{matrix}$ (S76)

This implies that the $a_{i}$ derivatives in (27) are

$\begin{matrix} \frac{\partial V}{\partial a_{1}}=a^{2}\pi\int_{\nu_{up}}^{\pi} 3\sinh^{2}\mu\sin^{2}\nu_{0}\left(\cosh\mu\sin\nu_{0}-\sinh\mu\cos\nu_{0}\frac{\partial\mu}{\partial\nu_{0}}\right) \\ +a(3\sinh^{3}\mu+2\sinh\mu)\sin^{3}\nu_{0}\frac{\partial\mu}{\partial a_{1}}-3a\sinh^{2}\mu\cosh\mu\sin^{2}\nu_{0}\cos\nu_{0}\frac{\partial\mu}{\partial\nu_{0}}\frac{\partial\mu}{\partial a_{1}} \\ -a\sinh^{3}\mu\sin^{2}\nu_{0}\cos\nu_{0}\frac{\partial}{\partial a_{1}}\left(\frac{\partial\mu}{\partial\nu_{0}}\right)d\nu_{0} \end{matrix}$ (S77)

$\begin{matrix} \frac{\partial V}{\partial a_{2}}=a^{3}\pi\int_{\nu_{up}}^{\pi} (3\sinh^{3}\mu+2\sinh\mu)\sin^{3}\nu_{0}\frac{\partial\mu}{\partial a_{2}}-3\sinh^{2}\mu\cosh\mu\sin^{2}\nu_{0}\cos\nu_{0}\frac{\partial\mu}{\partial\nu_{0}}\frac{\partial\mu}{\partial a_{2}} \\ -\sinh^{3}\mu\sin^{2}\nu_{0}\cos\nu_{0}\frac{\partial}{\partial a_{2}}\left(\frac{\partial\mu}{\partial\nu_{0}}\right)d\nu_{0} \end{matrix}$ (S78)

where $\mu$ is evaluated on the endocardium, i.e.$\mu=\mu{}_{in}(\nu_{0})$.

**Numerical solution method**

The model can be summarized as a system of five nonlinear differential algebraic equations (30, 33, S75) of the form

where $\mathbf{Y}=\left[\begin{matrix} a_{1} & a_{2} & a_{3} & P_{A} & P_{AO} \end{matrix}\right]^{T}$ (S76)

Since this system is a coupled set of nonlinear algebraic equations of $\mathbf{Y}$ and , standard differential equation solvers cannot be applied directly. Instead, we use a Newton iteration to solve the algebraic problem and subsequently time step using a standard solver. To illustrate this method, suppose that the current state of the system $\mathbf{Y}$ is known at some time step $t$ (either from initial conditions or a previously computed numerical solution). We want to solve for the form

(S77)

which allows the use of standard numerical methods. To obtain this form of the equation we combine the differential equations for the left ventricle (S75) and the relationships (30) and (33) to obtain the system

$\left\{\begin{matrix} \alpha_{1}\frac{da_{1}}{dt}+\beta_{1}\frac{da_{2}}{dt}+\gamma_{1}\frac{da_{3}}{dt}+\kappa_{1}-\eta_{1}p & =0 \\ \alpha_{2}\frac{da_{1}}{dt}+\beta_{2}\frac{da_{2}}{dt}+\gamma_{2}\frac{da_{3}}{dt}+\kappa_{2}-\eta_{2}p & =0 \\ \alpha_{3}\frac{da_{1}}{\partial t}+\beta_{3}\frac{da_{2}}{dt}+\gamma_{3}\frac{da_{3}}{dt}+\kappa_{3} & =0 \\ \frac{\partial V}{\partial a_{1}}\frac{da_{1}}{dt}+\frac{\partial V}{\partial a_{2}}\frac{da_{2}}{dt}-\frac{P_{A}-p}{R_{MV}}+\frac{p-P_{AOV}}{R_{AOV}} & =0 \\ P_{AOV}\left(R_{AO}+R_{AOV}\right)-pR_{AO}-P_{AO}R_{AOV} & =0 \end{matrix}\right.$ (S78)

This set of equations can be viewed as an algebraic system of the form

$\mathbf{h}(\mathbf{X})=0$ where (S79)

The values of the integrals and derivatives not included in $\mathbf{X}$ can be computed from the known values of $\mathbf{Y}$ and $t$, which implies that the variable $\mathbf{X}$ can be solved for by Newton iteration. The system (S78) is only nonlinear in the last two equations, so provided that the time steps are not too large the Newton iteration converges quickly. Once $\mathbf{X}$ has been computed, the system is in the form

where $\mathbf{g}(\mathbf{Y},t)=\left[\begin{matrix} X_{1} & X_{2} & X_{3} & g_{4} & g_{5} \end{matrix}\right]^{T}$ (S80)

The values of $g_{4}$ and $g_{5}$can be computed directly from $\mathbf{Y}$ using equations (29) and (30). We then solve for the approximate values of $\mathbf{Y}$ at the new time step using an RK2 method, which provides a mean between computational efficiency and stability. At the intermediate step of the RK2 method $\mathbf{g}(\mathbf{Y},t)$ must be recomputed by Newton iteration.
